# Health Resorts

**Published:** 1903-01-03

**Authors:** 


### HEALTH RESORTS.

### PLYMOUTH.

IT may seem strange to some to speak of a great bustling port like Plymouth as a health resort. Yet in practice it not unseldom happens that it is easier to find a suitable climate than to find a town in such a climate suitable to the particular patient whose case one is considering at the moment, and Plymouth even as a health resort has its place. There are plenty of people who will put up with the country for a time, and will gladly go and stay in country house amid country interests, but to whom the petty interests of a little town spell boredom thickly spread. To such, a sentence of banishment for the winter to some little seaside place, warm and cosy though it be, is a penance not lightly to be undergone. In the winter season especially numerous comforts which are to be had in large towns are quite inaccessible in smaller places, and there is no doubt that there are many people, not very ill perhaps, but whose health demands a warm and fairly stable winter climate, who would do well to think of Plymouth or its outskirts as a health resort. Of course there are great storms blowing up from the Atlantic, and, of course, there is a good deal of rain; not, it is stated, so many rainy days as in some other districts, but heavy rain by which the place is washed and the air purified. Warm though it may seem in the winter compared with northern and more easterly towns, Plymouth must not be regarded as a relaxing place, that is if one lives in the higher suburbs rather than in the town itself; while within what one may term excursion range there are many most interesting places which will be found well worthy of being visited when the weather enables the semi-invalids of whom we are thinking to run off for a day or two from their winter home. As a summer tourist centre Plymouth is well known; as a health resort it is anxious to be known; and there can be no doubt that as a climatic station it has its uses.

